# AP-XPS beamline, a platform for *operando* science at Pohang Accelerator Laboratory

**DOI:** 10.1107/S160057751901676X

**Published:** 2020-01-28

**Authors:** Geonhwa Kim, Youngseok Yu, Hojoon Lim, Beomgyun Jeong, Jouhahn Lee, Jaeyoon Baik, Bongjin Simon Mun, Ki-jeong Kim

**Affiliations:** a Department of Physics and Photon Science, Gwangju Institute of Science and Technology, Gwangju 61005, Republic of Korea; b Advanced Nano-Surface Research Group, Korea Basic Science Institute, Daejeon 34133, Republic of Korea; c Beamline Research Division, Pohang Accelerator Laboratory, Pohang 37674, Republic of Korea

**Keywords:** AP-XPS, SPEM, beamline, PAL (PLS-II)

## Abstract

Beamline 8A (BL 8A) is an undulator-based soft X-ray beamline at Pohang Accelerator Laboratory. This beamline is aimed at high-resolution ambient-pressure X-ray photoelectron spectroscopy (AP-XPS), soft X-ray absorption spectroscopy (soft-XAS) and scanning photoemission microscopy (SPEM) experiments. BL 8A has two branches, 8A1 SPEM and 8A2 AP-XPS, that share a plane undulator, the first mirror (M1) and the monochromator. The photon beam is switched between the two branches by changing the refocusing mirrors after the monochromator. The acceptance angle of M1 is kept glancing at 1.2°, and Pt is coated onto the mirrors to achieve high reflectance, which ensures a wide photon energy range (100–2000 eV) with high resolution at a photon flux of ∼10^13^ photons s^−1^. In this article, the main properties and performance of the beamline are reported, together with selected experiments performed on the new beamline and experimental system.

## Abstract 2

An ambient-pressure photoemission spectroscopy beamline has been constructed and has recently begun regular user service at Pohang Accelerator Laboratory. This beamline is a soft X-ray beamline with undulator (U6.8) insertion device and will be a platform for *operando* surface science.

## 1. Introduction   

Currently, scientists and engineers are searching for possible solutions to mitigate serious imminent global issues such as climate change, deficit energy resource and air pollution. These issues seem to be independent, yet they are closely interconnected. Solutions can be found from various fields of science and engineering, for example environmental science, catalysis, nanotechnology and energy technology, and, in particular, material science can provide a starting platform for any possible solution.

In material science, identifying or modifying the ‘surface/interface’ of the materials is the most important process as most physical or chemical reactions take places at the surface or interfaces (Ertl, 2015 ▸). While many analytical techniques are developed for studying the surface/interface, electron- or ion-based techniques provide highly surface sensitive information due to their short inelastic mean free path, *e.g.* ion scattering, secondary ion mass spectroscopy and X-ray photoemission spectroscopy. These tools have proven to be highly effective in studying the gas/solid surface (or interface) under ultrahigh vacuum (UHV) conditions. However, in the case of the liquid/solid surface (or interface) or the gas/solid interface under elevated pressure conditions, electron- or ion-based tools cannot be used.

In fact, since the early days of surface science, there has been a huge amount of effort in adapting UHV-based surface-science techniques to ambient-pressure (AP) systems (Siegbahn, 1969 ▸; Somorjai, 1978 ▸; Joyner *et al.*, 1979 ▸). Any surface chemical reaction mechanism occurring under UHV can differ significantly from that taking place under ambient-pressure conditions. As *in situ* surface studies have become increasingly important over the years, a number of innovative *operando* spectroscopy and microscopy techniques have also been developed (Shen, 1989 ▸; Hu *et al.*, 1995 ▸; Hansen *et al.*, 2001 ▸; Hendriksen & Frenken, 2002 ▸; Donald, 2003 ▸; Hüfner, 2003 ▸; Ferrer *et al.*, 2007 ▸; Forsberg *et al.*, 2007 ▸). Ambient-pressure X-ray photoemission spectroscopy (AP-XPS) is one of the prominent techniques that can provide surface/interface information under near ambient pressure conditions, *i.e.* in the ∼mbar range (Siegbahn, 1969 ▸; Joyner *et al.*, 1979 ▸; Ruppender *et al.*, 1990 ▸; Ogletree *et al.*, 2002 ▸; Salmeron & Schlögl, 2008 ▸). AP-XPS, built with differential pumping electrostatic lens system schemes, has clearly made a big contribution to the *operando* science community (Bluhm, 2010 ▸).

X-ray photoemission spectroscopy (XPS), the predecessor of AP-XPS, has been demonstrated as an invaluable technique in the study of filled electronic states of solids, as well as helping to determine the nature of interactions between solid surfaces and molecular species. But there is one main barrier of the technique – that XPS measurements on clean surfaces for surface science are required to be performed under UHV conditions (∼10^−10^ mbar). This limitation was partially overcome with the development of differentially pumped AP-XPS systems, which began in the early 1970s (Siegbahn & Siegbahn, 1973 ▸). Despite much effort, they suffered from poor electron yields due to the scattering of electrons in the gas phase. Then, with advances in electrostatic lens systems coupled with differential pumping of electron analyzer and high-flux synchrotron radiation sources, AP-XPS measurements became possible in the 100 mbar pressure range. AP-XPS has been recognized by scientific communities as an important *in situ* tool to study water, environmental science, catalysis and many other important fields (Salmeron & Schlögl, 2008 ▸; Starr *et al.*, 2013 ▸; Shavorskiy *et al.*, 2014 ▸). Since 2000, AP-XPS systems have been continuously installed at most major synchrotron radiation facilities around the world: ALS, BESSY, SSRL, MAX IV, SLS, ALBA, SOLEIL, SPring-8, Diamond, SSRF, and so on. Using these systems, many researchers have already published meaningful results in various fields (Artiglia *et al.*, 2017 ▸; Timm *et al.*, 2018 ▸; Kim *et al.*, 2018 ▸; Yu *et al.*, 2019 ▸; Soler *et al.*, 2019 ▸; Diulus *et al.*, 2019 ▸; Tesch *et al.*, 2019 ▸; Cai *et al.*, 2019 ▸).

Recently, beamline 8A (BL 8A) at Pohang Accelerator Laboratory (PAL) has been successfully reconstructed and an AP-XPS end-station is installed. Since 1999, BL 8A, the first undulator beamline at PAL, has been dedicated for surface/interface and material science by providing scanning photoemission microscopy (SPEM) (8A1) and high-resolution photoemission spectroscopy (HR-PES) (8A2) (Shin *et al.*, 1999 ▸). The undulator at BL 8A was designed to produce photon energies in the range 50–2000 eV, and BL 8A has been one of the most popular beamlines at PAL as a user-friendly end-station that can provide both microscopic and spectroscopic techniques for surface/interface science. However, the practically available photon energy range at the end-station was gradually reduced to 100–800 eV as beamline components including the optical system went out of exhaustive operation. User groups in surface/interface and material science have made continuous requests to recover the higher energy above 2000 eV and introduce an additional advanced photoemission method, *e.g.* AP-XPS, for *operando* science. Finally, in December 2014, PAL and KBSI signed a Memorandum of Understanding for the construction of a new beamline and AP-XPS experimental system to initiate a joint research program in emerging fields of science.

In this paper, we outline the main properties and performance of the beamline as characterized during commissioning at the PAL storage ring (PLS II). For fertilizing new *operando* science, the beamline was designed with a wide photon energy range (100–2000 eV) with high photon resolution. A high photon flux, ∼10^13^ photon s^−1^, was achieved by keeping the M1 mirror acceptance angle glancing at 1.2° as well as minimizing the number of total reflecting mirrors, four for the 8A1 SPEM branch and six for the 8A2 AP-XPS branch. We will also discuss the experimental capabilities and present a few showcase experiments which have been performed on the new beamline.

## 2. Beamline overview   

### 2.1. Photon source for BL 8A   

The U6.8 undulator for BL 8A receives electron bunches of 3 GeV and 400 mA from the storage ring of the PAL synchrotron (Shin *et al.*, 2013 ▸; Hwang *et al.*, 2014 ▸). The electron velocity is 0.9999999963733*c* and the Lorentz factor γ, given as γ = 1/(1 − *v* ^2^/*c* ^2^)^1/2^, is 11742. A total of 48 permanent magnets are displaced with a periodicity of 6.8 cm and the total length of the undulator is 3.3 m. The mechanically allowed gap between the upper and lower magnet arrays is from 16 to 90 mm, for which the deflection parameter *K* of the undulator is from 5.769 to 0.105, given by the following equation,
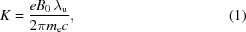
where *B*_0_ is the magnetic field at the centre of the undulator, *m*_e_ is the electron mass and λ_u_ is the period of the permanent magnets in the undulator. *B*_0_ is a function of the gap and the geometry of the undulator.

**Figure 1:**
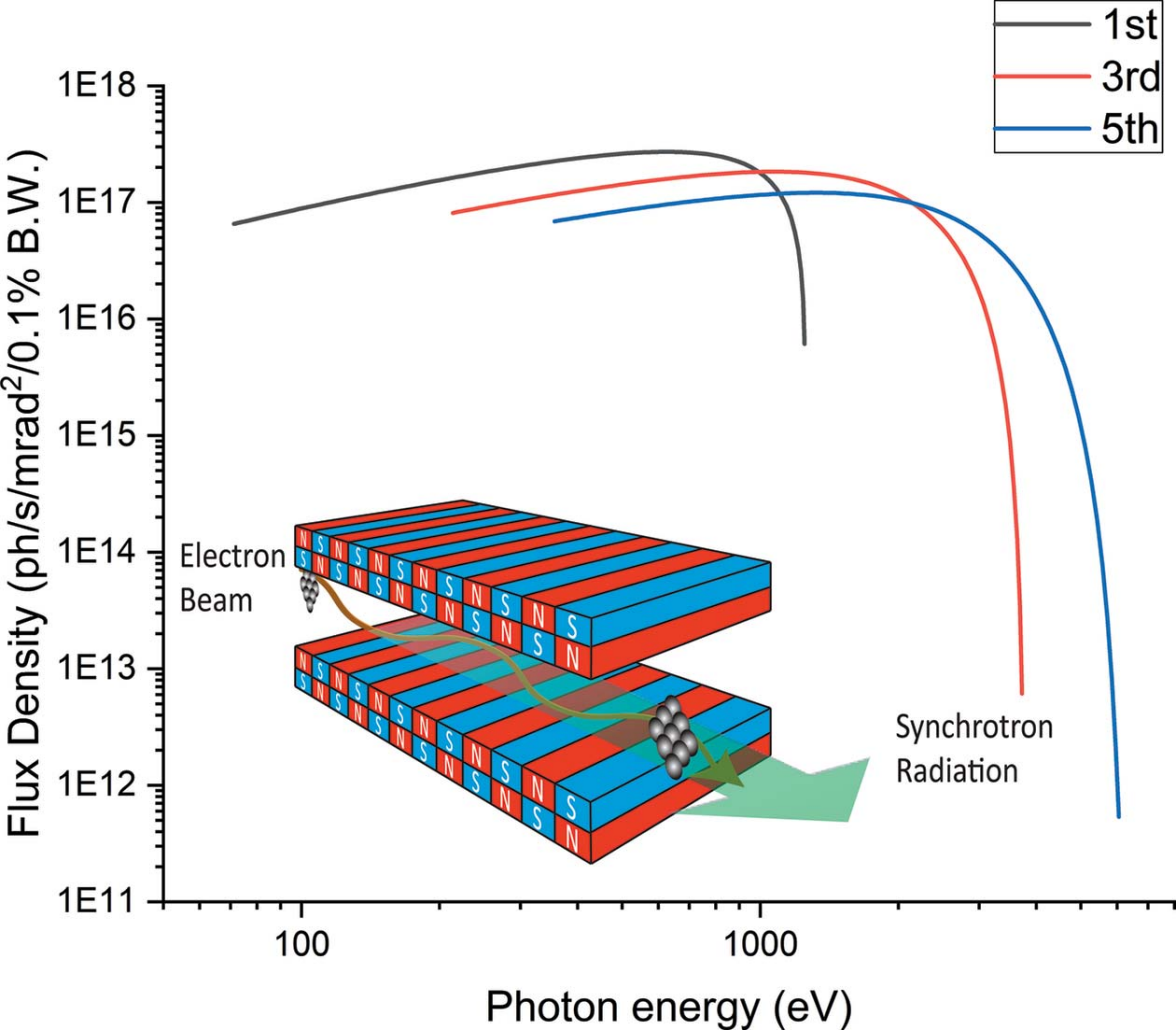
Calculated photon flux density of the U6.8 undulator radiation. The lines coloured black, red and blue represent the first, third and fifth harmonics, respectively. The inset shows a schematic diagram of the undulator.

With the given value of the parameter *K*, the photon energy *E* of the *n*th harmonics, which is radiated with emission angle θ referred to the beam propagating direction, is determined by the following equation,
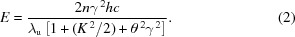
As a result of equation (2), the U6.8 undulator covers the energy range from 71.236 eV to 1248.94 eV for the first-harmonic radiation. Including the third and fifth harmonics, the undulator can cover the entire energy range that the beamline was designed for, *i.e.* 100–2000 eV, with a flux density of 10^17^ photons s^−1^ mrad^−2^ (0.1% bandwidth)^−1^ theoretically as shown in Fig. 1 ▸. The radiated beam is linearly polarized.

### 2.2. Beamline specifications   

**Figure 2:**
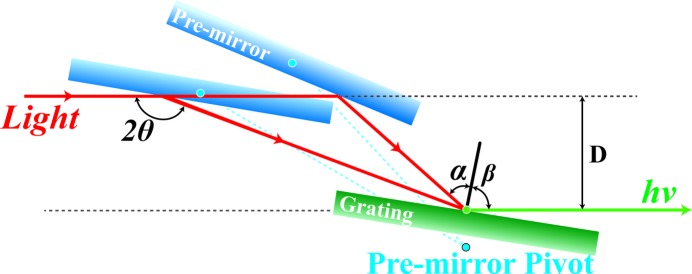
Schematic drawing of the operation mechanism of the PGM. The grating module rotates around the green pivotal point and the centre of the pre-mirror rotates around the blue pivotal point close to the grating, which always transfers white light to the centre of the grating.

The beamline optics are designed to accommodate the needs of the SPEM and AP-XPS end-stations, providing X-rays over a wide energy range from 100 to 2000 eV. The undulator can operate in the energy range from 70 eV up to 3000 eV, which corresponds to operation up to the third harmonic. The photon beam is collimated vertically by the first M1 mirror and delivered to the monochromator. The incident angle on the M1 mirror is 1.2° to ensure high reflectance at higher energy (∼1700 eV). The plane-grating monochromator (PGM) system is designed with SX-700-based optics to provide X-rays over a wide energy range (Petersen & Baumgärtel, 1980 ▸). Fig. 2 ▸ shows the conceptual design of the PGM. The operation of the monochromator follows the grating equation described by 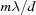 = 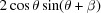, where *m* is the diffraction order, λ is the photon wavelength and *d* is the line spacing corresponding to the ruling density of the grating. *D*, the vertical distance offset between the entering and exiting X-ray paths, is 15 mm. By having a pre-collimated beam on the monochromator from the M1 mirror along the vertical direction, the fix constant, *C*_ff_, is allowed to vary freely. *C*_ff_ is given by *C*_ff_ = cosβ/cosα, and 2θ, the variable inclusive angle, is given by 2θ = α − β, where α > 0, β < 0 and 2θ are shown in Fig. 2 ▸. This additional flexibility of *C*_ff_ enables the monochromator to be optimized for enhanced flux and improved energy resolution.

Two different gratings are installed in a single monochromator unit, where the low-energy grating (LEG) is for the photon energy range 100–1100 eV, and the high-energy grating (HEG) is for the photon energy range 900–2000 eV. The LEG is ruled with a 400 lines mm^−1^ line density and 16 nm groove depth, and HEG is ruled with a 500 lines mm^−1^ line density and 8 nm groove depth.

The diffracted beam from the monochromator is focused onto the exit slit and switched by inserting the M3-1 (SPEM, 8A1) or M3-2 (AP-XPS, 8A2) toroidal mirror. As a result, the monochromatic beam after the M3 mirrors focuses onto the exit slit with a beam size of 210 µm (horizontal, H) × 5.9 µm (vertical, V) for 8A1 and 280 µm (H) × 7.1 µm (V) for 8A2. The aperture size of the exit slit can be determined by controlling four-way blades (*x*–*y*). Finally it is designed to achieve an energy resolving power of more than 5000 in the energy range that each grating can provide, when the vertical aperture size is kept at 40 µm, and LEG and HEG are operated with *C*_ff_ = 2.5 and *C*_ff_ = 1.7, respectively, with negative diffraction order.

**Figure 3:**
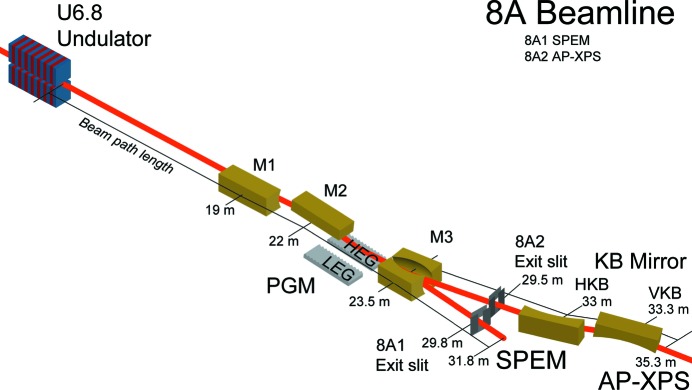
Optical layout and design of BL 8A. From the undulator, M1 horizontal focusing mirror, monochromator system with M2 and gratings, M3 and M3′ branching mirror for SPEM and AP-XPS, respectively, and exit slits and KB mirror system for AP-XPS are located.

In the SPEM (8A1) beamline, this monochromatic beam passes through a Fresnel zone plate and reaches the sample with a beam size of 100 nm. In the AP-XPS (8A2) beamline, Kirkpatrick–Baez (KB) mirrors, located at the end of the photon transfer line, tightly refocus the monochromatic beam onto the AP-XPS sample position. The FWHM of the beam size at the focal spot of the AP-XPS sample is less than 50 µm (V) × 50 µm (H). The beamline’s optical layout and design are schematically shown in Fig. 3 ▸. The optical and geometrical specifications of each optical element are listed in Table 1 ▸.

## 3. Beamline performance: photon resolution and photon flux   

**Figure 4:**
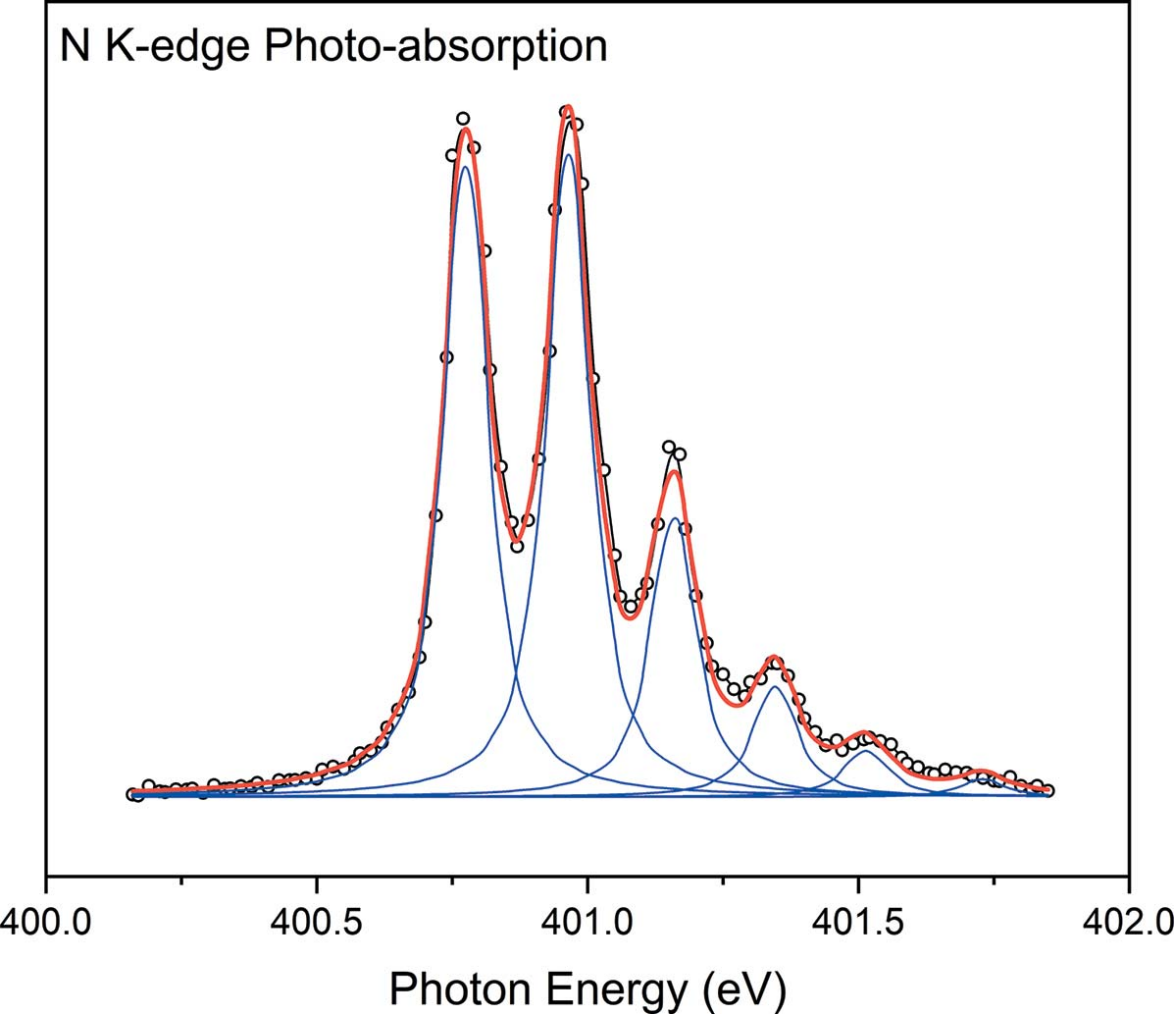
Photo-absorption spectrum of the nitro­gen *K*-edge taken under 10^−5^ mbar N_2_ gas pressure. The best deconvolution using a Voight function allows an 80 meV Lorentzian width and 40 meV Gaussian width.

Fig. 4 ▸ shows the nitro­gen *K*-edge absorption spectrum of gas-phase N_2_, which indicates the resolving power of the grating. The measurement was taken using a gas cell located between the exit slit and the KB mirrors of beamline 8A2 with an exit slit opening of 40 µm (V) × 200 µm (H). The gas cell was composed of two parallel plates and filled with N_2_ gas at a pressure of 10^−5^ mbar. One of the plates is biased by +100 V and pushes photo-ionized N_2_ molecules, and the other detects the collision of ions to itself by measuring current outputs.

The spectrum was deconvoluted with a Voigt function for which the Lorentzian and Gaussian widths were 80 meV and 40 meV, respectively. Since the Lorentzian width originates from lifetime broadening, the smaller value for this beamline compared with those of other soft X-ray beamlines (Lee & Shin, 2001 ▸; Watanabe *et al.*, 2004 ▸) can be due to using lower N_2_ gas pressure and providing fewer collision opportunities between ions and electrons. Additionally, the low signal-to-noise ratio of these spectra may come from the low N_2_ gas pressure. At 400 eV photon energy, the resolving power *E*/Δ*E* is estimated to be 400 eV/40 meV = 10000, which matches precisely the designed value of the LEG.

Also, the photon flux at the end-station, which will be used as an important value in actual experiments, was measured using a silicon photodiode (IRD 100G) located right after the exit slit for the 8A1 SPEM end-station (Gullikson *et al.*, 1996 ▸). In the most frequently used energy range of 100–2000 eV in both beamlines, the photon flux could be estimated as ∼10^13^ photons s^−1^.

## 4. Experimental station   

### 4.1. Windowless aperture system with differential pumping stages: buffering the pressure gap between the beamline and the experimental chamber   

The main obstacle in performing AP-XPS experiments is due to the elevated pressure in the main chamber in which experiments are conducted, which conflicts with the UHV requirement of the beamline. In many cases, a photon-transparent window, *e.g.* silicon nitride (Si_3_N_4_) or aluminium membrane (Al, thickness of ∼100 nm), has been installed between the beamline and AP-XPS experiment chamber to maintain the UHV condition of the beamline. BL 8A2, however, adopts a windowless differential pumping station (SPECS, Germany), installed between the experimental system and beamline with an aperture system with 200 µm-diameter capillary. Using four pumping stages and four apertures (with diameters of 6 mm, 5 mm, 4 mm and 3 mm in order from upstream), the pressure at the KB-mirror chamber can be maintained at ∼10^−9^ mbar.

### 4.2. Experimental components in AP-XPS   

**Figure 5:**
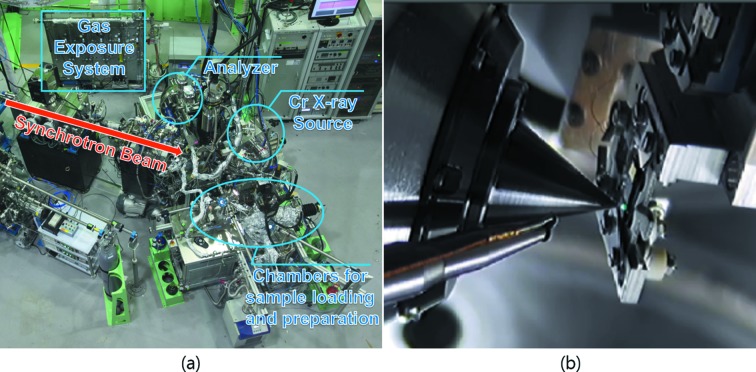
(*a*) Main experimental components of the AP-XPS end-station. (*b*) Beam spot site inside the analysis chamber. The angle between the analyser cone and beamline is 55° and the angle between the analyser cone and the Cr X-ray source is 79°.

The 8A2 AP-XPS end-station is aimed at surface studies under near ambient pressure conditions (up to 25 mbar, a pressure where liquid-phase water can exist at room temperature) as well as UHV conditions. The end-station consists of preparation and analysis chambers with two manipulators: one for preparing a well defined sample surface in UHV conditions and the other for AP-XPS measurements, as can be seen in Fig. 5(*a*) ▸. The preparation chamber includes the usual equipment for cleaning and annealing surfaces (Ar^+^ ion sputter gun and e-beam heater) and characterization of surface structure (LEED optics). There is a load-lock chamber for transferring the sample into the UHV chamber without venting the whole system. The analysis chamber, which has a backfilling configuration, is equipped with a SPECS Phoibos NAP 150 hemispherical analyzer for XPS. A Cr *K*α X-ray source (*h*ν = 5.417 keV) is also installed in the main chamber as an auxiliary photon source, and allows the bulk property to be analyzed with a higher probing depth by detecting photoelectrons with high kinetic energy. On the sample manipulator in the analysis chamber there are three sample stages for cooling down the sample using liquid nitro­gen, annealing the sample using an IR laser (Ostec, up to 1500 K) and direct-current heating of semiconductor samples, respectively. Fig. 5(*b*) ▸ shows the sample configuration inside the sample analysis chamber. Brief information on the AP-XPS experimental system is summarized in Table 2 ▸.

### 4.3. Probing depth change of SiO_2_/Si on photon energy   

**Figure 6:**
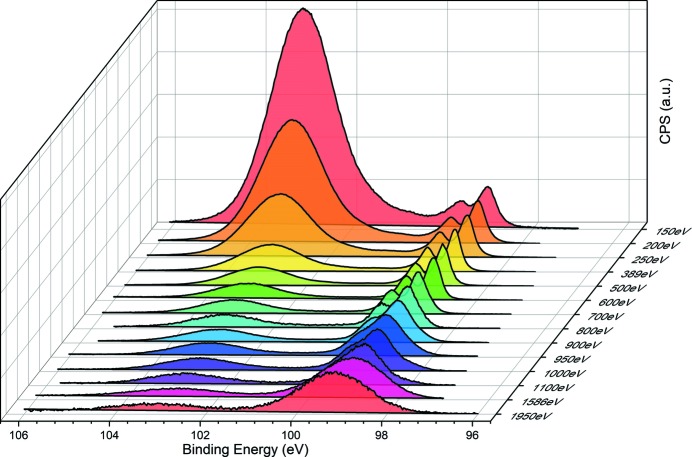
Si 2*p* spectra corresponding to photon energies from 150 eV to 1950 eV. The spectra are normalized with respect to the intensity of the Si 2*p*_3/2_ peak. For the LEG, the energy region from 150 eV to 1100 eV is covered, while the HEG covered 1586 eV to 1950 eV.

To test the beamline photon energy and the analyzer, XPS measurements were carried out on a SiO_2_/Si (100) sample for various photon energies. Fig. 6 ▸ shows the Si 2*p* XPS spectral changes for various photon energies under UHV conditions. For ease of comparison and avoidance of the difference by photoionization cross section, the spectra are normalized by the maximum intensity of the bulk Si 2*p*_3/2_ peak. Since the natural oxide was already formed on the surface, the oxide peak of Si 2*p*, whose intensity varies with photon energy, follows the typical escape depth curve of an electron. Using a photon energy of 150 eV to 1100 eV for the LEG, all the data points were collected only once with a 0.1 s dwell time and 20 meV step size at the most optimized position. The pass energy was 2 eV from 150 eV to 700 eV and 10 eV from 800 eV to 1100 eV. When the Si 2*p* core-level was measured using photon energies of 1586 eV and 1950 eV, for the HEG, the accumulation number is increased to overcome the reduced photon flux by five times and ten times, respectively, with 0.1 s dwell time and 50 eV pass energy. In summary, we would like to demonstrate the practical photon energy range by measuring the Si 2*p* core-level on SiO_2_/Si (100) and prove that the available photon energy range is 100–2000 eV at BL 8A.

### 4.4. Intensity change of Au 4*f*_7/2_ under N_2_ environments   

**Figure 7:**
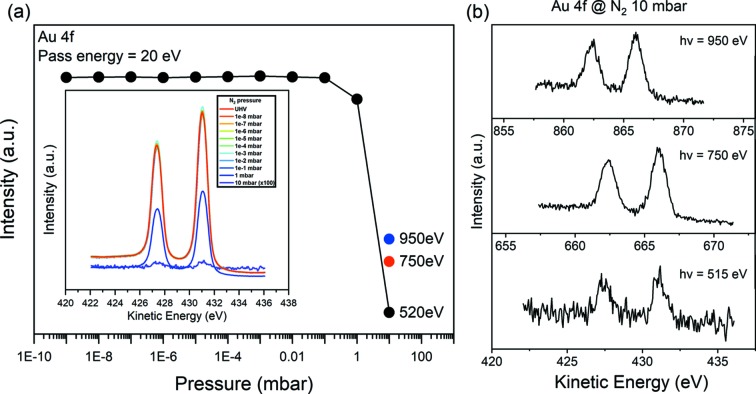
(*a*) Au 4*f* intensity change at elevated N_2_ pressure. The black dots show the maximum intensities of Au 4*f*_7/2_ peaks acquired from the spectra in the inset of (*a*). (*b*) Raw spectra of Au 4*f* taken under a 10 mbar N_2_ gas environment. All the spectra are collected in one scan.

A simple performance test of AP-XPS was carried out with an Au reference. Fig. 7(*a*) ▸ shows the intensity change of Au 4*f*_7/2_ for a gradual change of the N_2_ pressure at a photon energy of 520 eV. All of the spectra in Fig. 7 ▸ are acquired with a 0.1 s dwell time, 50 eV pass energy, 50 meV step energy, 3 mm × 25 mm analyzer slit and 40 µm (V) × 200 µm (H) 8A2 exit slit. All signals are normalized using the beam current at the KB mirror. As the pressure of N_2_ is varied from 10^−9^ to 0.1 mbar, Au 4*f*_7/2_ has almost the same intensity. At 1 mbar, the signal intensity starts to decrease and reduces rapidly in the 1–10 mbar range. At 10 mbar, the signal is very low due to the short inelastic mean free path of the photo-electrons which cannot overcome the distance between the sample and the nozzle of the spectrometer. In practice, signal is collected sufficiently to distinguish components in the spectrum up to 7–8 mbar in the case of N_2_ gas for photon energies of the LEG. Fig. 7(*b*) ▸ shows the Au 4*f*_7/2_ spectra at photon energies of 750 eV and 950 eV at 10 mbar. It shows an increased intensity because of the prolonged inelastic mean free path of the electrons at higher photon energy.

### 4.5. Photo-induced nitro­gen doping on graphene/Ge at near ambient pressure   

**Figure 8:**
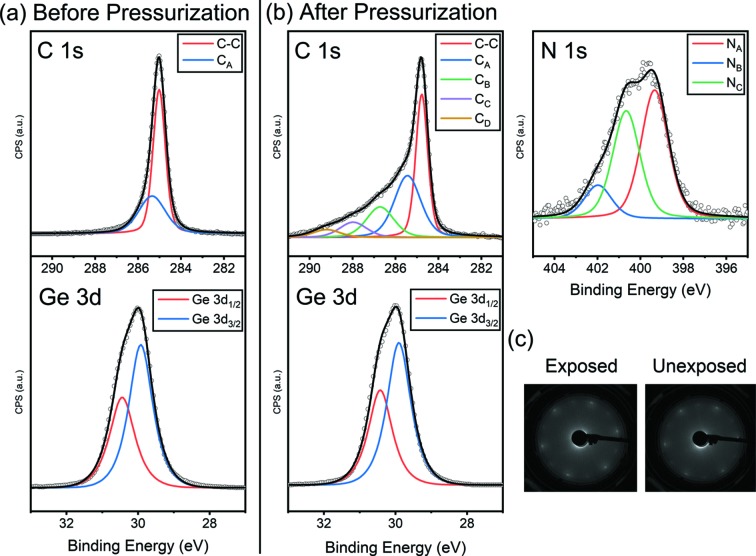
UHV states of exposed graphene/Ge (110) (*a*) before and (*b*) after pressurization, and (*c*) LEED patterns of final N 1*s* states of exposed and unexposed graphene/Ge samples to the synchrotron beam. N-related species are produced immediately after arrival at the 10 mbar condition under the irradiation. All the spectra are taken at 700 eV photon energy and 50 eV pass energy.

An investigation of the photo-induced effect during the N_2_ gas pressurization onto graphene/Ge is a good example of AP-XPS. The graphene on Ge (110) substrate system has been reported as a good stage for dry-transferring of graphene (Kim *et al.*, 2013 ▸; Yang *et al.*, 2019 ▸), but many of its properties are not yet characterized. For the investigation, two identical graphene/Ge samples are prepared as previously reported (Yang *et al.*, 2019 ▸), and each is located at different stages in the main chamber manipulator to compare the effects of photon irradiation by exposing only one of them to a synchrotron beam (*h*ν = 700 eV) during the pressurization. N_2_ gas pressure was controlled from UHV to 10 mbar to trace the generation of nitro­gen species on the surface of the sample, and then pumped out to UHV again. By that process, the same atmospheric conditions are applied to both samples. During the whole experiment, no heat or other gases are applied on the samples. Fig. 8 ▸ shows the fitting results of C 1*s*, Ge 3*d* and N 1*s* spectra of the exposed sample measured at UHV before and after pressurization, and the LEED patterns of the exposed and unexposed samples.

As shown in Fig. 8(*b*) ▸, several components of N 1*s* at binding energy 399.3 eV (N_A_), 400.7 eV (N_B_) and 402 eV (N_C_) were found after the pressurization only for the photon-irradiated sample and only for the irradiated spot. These components are not reduced or removed even by annealing or ageing in UHV. Simultaneously, C 1*s* of final states has an enhanced shoulder in the higher binding energy region compared with its initial state, shown in Fig. 8(*a*) ▸. On the other hand, Ge 3*d* shows no change during the process. Additionally, beam exposure did not affect the LEED pattern of graphene as shown in Fig. 8(*c*) ▸. Therefore, it can be inferred that nitro­gen atoms have some interaction with carbon atoms by irradiation but do not destroy the overall 2D structure of graphene. In previous investigations (Shao *et al.*, 2010 ▸; Usachov *et al.*, 2011 ▸; Mokhtar Mohamed *et al.*, 2018 ▸; Xu *et al.*, 2018 ▸), mainly three different types of nitro­gen bonding to carbon atoms are reported in the N-doped graphene: pyridinic, pyrrolic and graphitic bonding. Based on this, the enhanced shoulder of the C 1*s* peak could be deconvoluted into three peaks at binding energy 286.7 eV (C_B_), 288.0 eV (C_C_) and 289.2 eV (C_D_), and they might be coupled with N_A_, N_B_ and N_C_, respectively.

Since only the exposed sample has nitro­gen-related species during the pressurization, those components can be thought of as being produced by the photo-induced effect. This result is quite encouraging because it can be a new method to synthesize N-doped graphene. Further research will be conducted for more detailed information.

## 5. Summary   

The AP-XPS beamline offers a platform for *operando* experiments at UHV and ambient-pressure conditions. With the combination of a soft X-ray (100–2000 eV) and Cr X-ray source, two photon sources allows the electronic structure of matter to be studied from UHV to elevated pressures up to 25 mbar both at the surface and in the bulk. Beamline performance tests demonstrated that the beamline meets the design parameters very well. The estimated photon resolving power through the N *K*-edge is more than 10000, and the photon flux measured using an IRD photodiode is ∼10^13^ photon s^−1^. AP-XPS measurements of the Si and Au sample under elevated pressure also meet the expected performance while AP-XPS spectra of the graphene/Ge system under nitro­gen pressure demonstrated the presence of the photo-induced effect. The beamline and AP-XPS end-station are currently open to public users at the 3.0 GeV ring at PAL.

## Figures and Tables

**Table 1:** Beamline optics specifications

Name of optics	M1 mirror	M2 pre-mirror	Gratings	M3-1 (8A1) / M3-2 (8A2)	HKB (8A2)	VKB (8A2)
Position from source	19 m	22 m	23.5 m	22 m / 25 m	19 m	19 m
Shape	Cylindrical	Plane	Plane	Toroidal	Cylindrical	Cylindrical
			LEG: 400 l mm^−1^			
			HEG: 500 l mm^−1^			
Function	Vertical collimation	Just reflection	Diffraction	Focusing to exit slit	Horizontal refocusing	Vertical refocusing
Tangential radius	Infinite	Infinite	Infinite	32000 cm / 40000 cm	10000 cm	7550 cm
Sagittal radius	8012.3 mm	Infinite	Infinite	4400 mm / 3138 mm	Infinite	Infinite
Substrate material	Glidcop (internal cooling)	Glidcop (internal cooling)	Single-crystal silicon	Single-crystal silicon	Single-crystal silicon	Single-crystal silicon
Incident angle	1.2° (in and out)	Variable (0.9°–5.0°)	Variable (0.9°–5.0°)	2.0° / 1.5°	1.5° (in and out)	2.0° (in and out)
Coating thickness (Å)	Au (600 Å)	Au (600 Å)	Au (600 Å)	Au (600 Å)	Au (600 Å)	Au (600 Å)
Beam size @ 830 eV (µm)	1240 × 610	1330 × 610	1360 × 610	1370 × 4060 / 1370 × 4060	760 × 2370	570 × 2900
Footprint @ 830 eV (µm)	58400 × 606	1330 × 17600	1360 × 119900	49500 × 4060 / 54600 × 4060	29100 × 2370	570 × 78700

**Table 2:** Brief information for the AP-XPS experimental system

Electron analyzer	SPECS Phoibos NAP 150 hemispherical analyser with 2D delay line detector
Photon energy	100–2000 eV, 5417 eV (Cr X-ray source)
Pressure	UHV ∼25 mbar
Temperature control	Liquid nitro­gen (150 K)
	IR laser heater (up to 1500 K)
	Direct-current heating (up to 1500 K)
Gases	N_2_, O_2_, CO_2_, CO, Ar, Ne…
Equipment	Low-energy electron diffraction (LEED)
	Ion gun
	Electron beam heater
	Residual gas analyser (RGA)
Applicable research area	Physics at surface and interface
	Gas-phase heterogeneous catalysis
	Electrochemistry (battery, fuel cell, corrosion, electrocatalysis)
	Photovoltaic
	Environmental science
	Biological systems…
